# The association between dairy intake in adolescents on inflammation and risk markers of type 2 diabetes during young adulthood: results of the DONALD study

**DOI:** 10.1017/S1368980024000624

**Published:** 2024-03-13

**Authors:** Eva Hohoff, Nicole Jankovic, Ines Perrar, Maike Schnermann, Christian Herder, Ute Nöthlings, Lars Libuda, Ute Alexy

**Affiliations:** 1 Department of Nutritional and Food Sciences – Nutritional Epidemiology, University of Bonn, DONALD Study, Heinstück 11, Dortmund, Germany; 2 Institute for Clinical Diabetology, German Diabetes Center, Leibniz Center for Diabetes Research, Heinrich Heine University Düsseldorf, Düsseldorf, Germany; 3 German Center for Diabetes Research (DZD), Partner Düsseldorf, München-Neuherberg, Germany; 4 Department of Endocrinology and Diabetology, Medical Faculty and University Hospital Düsseldorf, Heinrich Heine University Düsseldorf, Düsseldorf, Germany; 5 Department of Sports and Health – Institute of Nutrition, Consum and Health – Nutritional Science, University of Paderborn, Paderborn, Germany

**Keywords:** Dairy, Inflammation, Insulin resistance, Children, Adolescents

## Abstract

**Objective:**

The aim of this analysis was to investigate whether habitual intake of total dairy (TD) or different dairy types (liquid, solid, fermented, non-fermented, low-fat, high-fat, low-sugar and high-sugar dairy) during adolescence is associated with biomarkers of low-grade inflammation as well as risk factors of type 2 diabetes in young adulthood.

**Design:**

Multivariable linear regression analyses were used to investigate prospective associations between estimated TD intake as well as intake of different types of dairy and a pro-inflammatory score, based on high-sensitivity C-reactive protein, IL-6, IL-18, leptin and adiponectin, and insulin resistance assessed as Homeostasis Model Assessment Insulin Resistance in an open-cohort study.

**Setting:**

Dortmund, Germany.

**Participants:**

Data from participants (*n* 375) of the DOrtmund Nutritional and Anthropometric Longitudinally Designed (DONALD) study were included, for whom at least two 3-d weighed dietary records during adolescence (median age: 11 years) and one blood sample in young adulthood (>18 years) were available.

**Results:**

There was no statistically significant association between TD intake or intake of any dairy type and the pro-inflammatory score (all *P* > 0·05). TD intake as well as each dairy type intake and insulin resistance also showed no association (all *P* > 0·05).

**Conclusions:**

The habitual intake of dairy or individual types of dairy during adolescence does not seem to have a major impact on low-grade systemic inflammation and insulin resistance in the long term. There was no indication regarding a restriction of dairy intake for healthy children and adolescents in terms of diabetes risk reduction.

In Germany and most Western countries, dairy intake is an essential part of a healthy diet for children and adolescents because of its nutrient composition that is beneficial for healthy growth (e.g. protein or Ca content)^(1,2)^. Nevertheless, dairy intake has been linked with both positive^(3–5)^ and potential negative effects on human health^(6–8)^. Systematic reviews and meta-analyses of randomised clinical trials analysing the relationship between dairy intake and low-grade systemic inflammation in adults showed neutral to beneficial effects on various inflammatory biomarkers^(9–13)^. In a systematic review, dairy intake was associated with anti-inflammatory activity among people with metabolic disorders and pro-inflammatory activity among subjects allergic to cow’s milk^(14)^. According to the authors, these opposing effects can be attributed to the hypersensitive reaction and the resulting pro-inflammatory state in subjects with bovine milk allergy. Apart from these subgroup-specific pathophysiological mechanisms, the heterogeneity of this specific food group could be relevant for metabolic effects. Based on the nutrient content (fat and sugar), processing methods (fermentation) or consistency (liquid and solid), different types of dairy may have different effects on metabolism^(15)^. In fact, the consideration of dairy types instead of overall dairy shows anti-inflammatory activity of low-fat, high-fat and fermented dairy intake^(14)^. Inflammatory processes have been identified as possible risk factors for obesity, insulin resistance, type 2 diabetes and CVD^(16,17)^. However, studies assessing the associations between different types of dairy and inflammatory biomarkers are scarce and have been mainly conducted in adults^(18)^. Evidence of a potential association of total dairy (TD) or dairy types in children and adolescents is limited, although this is a vulnerable group. Nevertheless, a recent meta-analysis examining the relationship between dietary intake and biomarkers of inflammation among healthy children and adolescents revealed that there is no association between dairy intake and inflammation markers, such as C-reactive protein or IL-6, in children and adolescents^(19)^. However, this meta-analysis included only a few studies focusing on the association with TD, and most of the included studies were cross-sectional. Although these cross-sectional studies have not shown that dairy intake as part of a healthy diet has unbeneficial associations with inflammation markers in children and adolescents in the short term^(19)^, no studies have analysed the long-term association from adolescence to young adulthood. Furthermore, whether this potential association differs with dairy types remains unknown.

Therefore, this study aimed to investigate the relationship between dairy intake as well as intake of different types of dairy in adolescence and biomarkers of inflammation and insulin resistance in young adulthood.

## Methods

### DONALD study

The DOrtmund Nutritional and Anthropometric Longitudinally Designed (DONALD) study is an ongoing dynamic (open) cohort study that collects information on nutrition, growth, development and metabolism of healthy children and adolescents in Dortmund, Germany. The study was initiated with a cross-sectional sample of children and adolescents (approximately 640 participants, >2 years old) recruited in 1985. Since 1985, 35–40 infants have been enrolled annually. Eligibility criteria are healthy infants whose parents are willing to participate in a long-term study and at least one parent with sufficient knowledge of German. Participants are initially examined at the age of 3 months and return for three more follow-up visits in the first year, two in the second year and then once annually until young adulthood. Annual examinations include 3-d weighed dietary records, anthropometric measurements, 24-h urine samples, lifestyle interviews and medical examinations. Parental examinations occur every 4 years. The study was non-invasive during childhood and adolescence. Since 2005, participants aged >18 years have been invited for subsequent examinations with fasting blood samples. Further details of the study have been described elsewhere^(20)^. This study was registered in the German Register of Clinical Trials (DRKS-ID: DRKS00029092).

### Study sample

In June 2019, 17 782 dietary records of 1706 children were available. Incomplete records (<3 d, *n* 98 records) were excluded. The inclusion criteria for the present analyses were as follows: all available data of participants who provided at least two 3-d dietary records (median number of individual 3-d dietary records: 8) during adolescence (girls, 8–15 years; boys, 9–16 years; median of all, 12 years) (Tables 1 and 2) and at least one fasting blood analysis in young adulthood (median age at blood sampling: 20·9 years). This resulted in samples from 375 participants for the current inflammation analyses. Of these, Homeostasis Model Assessment Insulin Resistance (HOMA2-IR) data were available for 371 participants, which was considered for the analyses on insulin resistance. The median follow-up of 9·2 (6·4, 12·5) years was defined as the number of years between the median age during adolescence and age during blood withdrawal.

**Table 1:** Sample characteristics of 375 participants of the DONALD study in adolescents: anthropometry, dietary data as well as early life, family and socio-economic factors

Sample characteristics for adolescence	*n*	Value	
		*n* or medians	% or 25th, 75th percentile
General characteristics			
Records ♂	2561	1·181	46·1
Participants ♂	375	174	46·4
Early-life factors			
Duration of gestation (weeks)	373	40	39; 41
Gestational weight gain (kg)	365	13	10; 15
Birth weight (g)	374	3435	3130; 3760
Full breast-feeding ≥4 months	375	266	71
Family characteristics			
Maternal BMI (kg/m^2^)*	371	24·03	21·89; 26·92
Maternal overweight†	371	145	39·1
Maternal employment	375	250	66·7
Descriptive data at dietary assessment			
Individual mean age (years)	2561	12·0	10·0; 14·0
Individual mean age (years) ♂	1181	12·3	10·3; 14·2
Individual mean age (years) ♀	1380	11·2	9·1; 13·2
Sleep duration (hours/night)	371	9·29	8·86; 9·75
Age at take-off‡ (years)	283	9·1	8·0; 10·4
Anthropometrics			
BMI-SDS||	373	0·11	–0·60; 0·67
FMI (kg/m²)¶	373	3·16	2·43; 4·69
FFMI (kg/m²)**	373	14·7	14·0; 15·8
Nutrition parameters			
*n* records in adolescents per participant	375	8	6; 8
Under-reporting†† participants	375	10	2·7
Total energy intake (kcal/d)	375	1870·57	1633·06; 2097·34
Dietary intake (g/d)	375	2106·92	1799·21; 2421·00
Total dairy intake (g/1000 kcal)	375	177·87	127·35; 232·60
Liquid dairy intake (g/1000 kcal)	375	78·61	40·84; 131·06
Solid dairy intake (g/1000 kcal)	375	86·52	63·28; 115·91
Non-fermented dairy intake (g/1000 kcal)	375	130·91	81·03; 184·31
Fermented dairy intake (g/1000 kcal)	375	44·85	28·08; 59·97
Low-fat dairy intake (g/1000 kcal)	375	35·56	14·99; 95·85
High-fat dairy intake (g/1000 kcal)	375	107·18	64·31; 166·50
Low-sugar dairy intake (g/1000 kcal)	375	124·53	76·67; 176·54
High-sugar dairy intake (g/1000 kcal)	375	46·92	30·03; 64·99

DONALD, DOrtmund Nutritional and Anthropometric Longitudinally Designed; BMI-SDS, BMI–SD score; FMI, fat mass index; FFMI, fat-free mass index.

Values are *n* (%) or medians (25th, 75th percentile).

* BMI, kg/m².

† BMI > 25 kg/m².

‡ Age at minimal height velocity at the onset of the pubertal growth spurt^(37)^.

|| BMI–SD score (based on the German reference percentiles for children and adolescents)^(34)^.

¶ FMI (the underlying percentage body fat was estimated using the equations of Slaughter)^(35)^.

** FFMI (the underlying percentage body fat was estimated using the equations of Slaughter)^(35)^.

†† Paediatric cut-off values for under-reporting^(42)^.

**Table 2:** Sample characteristics of 375 participants of the DONALD study in young adulthood: anthropometry, blood data and lifestyle factors

Sample characteristics for young adulthood	*n*	Value	
		*n* or medians	% or 25th, 75th percentile
Follow-up* (years)	375	9·2	6·4; 12·5
Descriptive data at blood withdrawal			
Age at blood withdrawal (years)	375	20·9	18·1; 24·1
Age at blood withdrawal (years) ♂	174	19·5	18·1; 23·6
Age at blood withdrawal (years) ♀	201	21·0	18·1; 24·1
Anthropometrics	375		
BMI (kg/m²)†	375	22·39	20·69; 25·00
Waist circumference (cm)	375	76·30	70·80; 82·20
FMI (kg/m²)‡	373	5·75	4·04; 7·21
Lifestyle in adulthood			
Smoker§	311	79	25·4
Alcohol consumer||	352	330	91·3
Currently doing sports¶	335	335	100
Currently employed**	217	205	94·5
Inflammatory biomarker			
hsCRP†† (mg/l)	375	0·80	0·40; 2·00
IL-6‡‡ (pg/ml)	375	0·67	0·46; 1·06
IL-18§§ (pg/ml)	375	248·75	203·54; 309·35
Leptin (µg/l)	375	7·19	2·89; 14·19
Adiponectin (µg/l)	375	7·333	5·056; 10·408
Pro-inflammatory-score	375	−0·065	–0·340; 0·258
Risk marker of type 2 diabetes			
HOMA2-IR||||	371	1·45	1·16; 1·85
Fasting glucose (mg/dl)	371	91	87; 97

DONALD, DOrtmund Nutritional and Anthropometric Longitudinally Designed; FMI, fat mass index; hsCRP, high-sensitivity C-reactive protein; HOMA2-IR, Homeostasis Model Assessment Insulin Resistance.

Values are *n* (%) or medians (25th, 75th percentile).

* Median age at adolescence and age at blood withdrawal.

† BMI, kg/m².

‡ FMI (the underlying percentage body fat was estimated using the equations of Durnin–Womersley)^(36)^.

§ Smoker included current and occasional smoking and was defined based on the variable ‘smoking (yes/no/sometimes)’.

|| Alcohol consumer was defined based on the variable ‘alcohol is currently consumed (yes/no)’.

¶ Currently doing sports (organised, not organised and no sport).

** Currently employed (yes/no/rest/in education).

†† hsCRP.

‡‡ IL-6, IL-6.

§§ IL-18, IL-18.

|||| HOMA2-IR.

### Dietary assessment

All food and beverages consumed and leftovers were weighed and recorded on 3 d by the parents or older participants themselves using electronic food scales. Semi-quantitative recording (e.g. spoons and cups) was allowed if accurate weighing was not possible. Information on recipes (ingredients and preparation) and types and brands of commercial food products were also required. Food group, energy and nutrient intake were calculated using our continually updated in-house food composition database LEBTAB^(21)^. The composition of basic foods was retrieved from the German food composition tables BLS 3.02. The energy and nutrient contents of commercial food products, that is, canned foods, ready-to-eat meals or snacks, were estimated by recipe simulation based on the listed ingredients and nutrients^(21)^.

According to Hohoff *et al.* ^(22,23)^, the following types of dairy were included in the analyses:

- TD

With regard to the nutrient content:

- Low- and high-fat dairy
- Low- and high-sugar dairy

With regard to the processing method:

- Fermented and non-fermented dairy

With regard to the way of intake:

- Liquid and solid dairy

Detailed descriptions of the different dairy types are given in Table 3. The daily intake of dairy and dairy types was calculated from the individual mean of all 3-d dietary records of participants examined during adolescence. To consider sex- and age-dependent differences in dietary intake, dairy intake was also standardised as g/1000 kcal of total energy intake.

**Table 3:** Classification of dairy products*, DONALD study^(23)^

	Included dairy products
Total dairy (TD)†	All dairy products (including dairy from cows and other mammals, such as goats or sheep)
Types of dairy	
Liquid dairy	Fresh milk, non-fermented and fermented drinks (e.g. cacao, buttermilk, whey), liquid sour milk products (including squeeze sour milk), yoghurt drink
Solid dairy	Non-fermented and fermented dairy food (e.g. yoghurt and cheese), milk for cereals or pudding
Fermented dairy	Fermented liquid and solid dairy: fermented dairy drinks (buttermilk and whey), liquid sour milk products (including squeeze sour milk), yoghurt drink, yoghurt, firm sour milk products, fermented desserts, fresh cheese, quark and cream fraiche, and cheese (soft cheese, sliced cheese, hard cheese and processed cheese)
Non-fermented dairy	Non-fermented liquid and solid dairy: milk, non-fermented dairy drinks (e.g. cocoa and milk shakes) and non-fermented milk desserts
Low-fat dairy‡	Non-fermented and fermented beverage dairy, non-fermented solid dairy, fermented solid dairy (fresh cheese, quark) <2 % fat, fresh cheese, quark (<9 % fat), soft cheese, processed cheese (<15 % fat), and semi-hard and hard cheese (<18 % fat)
High-fat dairy‡	Non-fermented and fermented beverage dairy, non-fermented solid dairy, fermented solid dairy (fresh cheese and quark) >2 % fat, fresh cheese, quark (>9 % fat), soft cheese, processed cheese (>15 % fat), and semi-hard and hard cheese (>18 % fat)
Low-sugar dairy§,||	Natural sugar content and added sugar <7 g/100 g industrially sweetened dairy
High-sugar dairy§,||	Added sugar >7 g/100 g industrially sweetened dairy

DONALD, DOrtmund Nutritional and Anthropometric Longitudinally Designed.

* Dairy products can occur in different groups.

† Excluding cream cakes and ice cream, because they are consumed as sweets rather than to meet dairy requirements, and excluding butter.

‡ Classification based on https://www.lebensmittellexikon.de/f0000170.php.

§ Including instant powders for milk (i.e. cocoa).

|| The cut-off was set based on the 1st quartile (6.9 g added sugar/100 g) from all sweetened dairy products (*n* 965) reported by the study sample.

### Blood analysis

Venous blood samples were drawn after an overnight fast, centrifuged at 4°C for 15 min and stored at –80°C in the DONALD study centre. Fasting plasma glucose levels were determined using a Roche/Hitachi Cobas c 311 analyzer. Plasma insulin concentrations were measured at the Laboratory for Translational Hormone Analytics of the University of Giessen using an immunoradiometric assay (IRMA, DRG Diagnostics). All other measurements were performed at the German Diabetes Center with the following assay characteristics^(24,25)^: plasma high-sensitivity C-reactive protein (hsCRP) with the Roche/Hitachi Cobas c311 analyser (Roche Diagnostics), plasma high-sensitivity IL-6 using the Human IL-6 Quantikine HS, plasma adiponectin with the Human Total Adiponectin/Acrp30 Quantikine ELISA, serum leptin with the Leptin Quantikine ELISA and serum IL-18 using the Human IL-18 ELISA kit from MBL.

### Definitions of the outcome variables

To estimate associations with low-grade chronic inflammation, a pro-inflammatory score was used, similar to that used by Diederichs *et al.* ^(24)^ and Penczynski *et al.* ^(26)^. This score is composed of established inflammation biomarkers that are assumed to reflect low-grade inflammation better than individual markers^(27)^. These include hsCRP, IL-6, IL-18, leptin and adiponectin. To approximate normal distribution, the individual biomarkers were log-transformed before standardisation (*z*-score) by sex (mean = 0, s d = 1). Then, these *z*-scores of the individual inflammation biomarkers were averaged, resulting in the pro-inflammatory score. Here, the anti-inflammatory parameter adiponectin was multiplied by −1.

Hence, the pro-inflammatory score was calculated as follows:

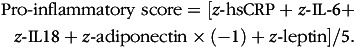




In addition, insulin resistance was assessed using the updated HOMA2-IR. HOMA2-IR is based on fasting insulin and blood glucose levels according to Wallace *et al.* ^(28)^:

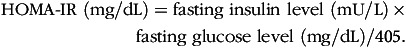




HOMA2-IR was also log-transformed before standardisation (*z*-score) by sex (mean = 0, s d = 1) to approximate normal distribution.

#### Assessment of potential confounding factors

Potential confounding factors were selected on the basis of the known predictors of low-grade systemic inflammation^(29,30)^ and type 2 diabetes^(31,32)^. At the first visit to the study centre, early-life factors such as pregnancy characteristics and birth anthropometry of the mother and child were taken through a standardised document (Mutterpass), which is issued to women during pregnancy in Germany. Anthropometric measurements (height, weight and skinfolds) were performed by trained nurses according to standard procedures using an electronic scale (Seca 753E; Seca Weighing and Measuring System, ±100 g), a digital stadiometer (Harpenden, ±0·1 cm) and a calliper (±0·1 mm, Holtain Ltd). The participants were dressed in underwear and were barefoot. The BMI (kg/m²) was calculated as the body weight (kg) divided by the square of the body height (m^2^). For adolescents, sex- and age-specific standard deviation scores were calculated based on the German reference percentiles for children and adolescents^(33)^. Percent body fat was estimated using the Slaughter^(34)^ and Durnin–Womersley equations^(35)^ for adolescents and adults, respectively. Body fat mass (kg) was calculated as [(percent body fat × body mass)/100]. The corresponding indices, fat mass index and fat-free mass index, were calculated by dividing the corresponding values by the square of the body height (m^2^). For adolescents and young adults, the respective medians were derived for all anthropometric data. In addition, parents were weighed and measured at regular intervals using the same equipment as used for the participants. Maternal overweight was defined as a BMI ≥ 25–<30 kg/m^2^ and maternal obesity as a BMI ≥ 30 kg/m^2^. In addition, parents were asked about their family and socio-economic characteristics (e.g. maternal education). Lifestyle factors of participants such as alcohol intake (yes/no), smoking (yes/no/sometimes), active in sports (organised/not organised/no sports) and employment (yes/no/rest/in education) were also collected by questionnaires.

The missing values were completed by the respective median of the total sample (pregnancy duration *n* 2; pregnancy weight gain *n* 10; birth weight *n* 1; maternal overweight *n* 4; adolescents’ BMI–standard deviation score, fat mass index and fat-free mass index *n* 2; sleeping duration in adolescence *n* 4; fat mass index in adulthood *n* 2; smoking status *n* 64; alcohol intake *n* 23; and physical activity *n* 40). The puberty status ‘age of take-off’, defined as the age at minimal height velocity at the onset of the pubertal growth spurt^(36)^, and ‘employment during adulthood’ could not be considered because of too many missing data (>20 %).

### Statistical analyses

All statistical analyses were performed using SAS^®^ procedures (version 9.20 and 9.40). The significance level was set at *P* < 0·05. Descriptive data are presented as median, with interquartile ranges for continuous variables and frequencies and percentages for categorical variables (Tables 1 and 2).

Multivariable linear regression was used to analyse the prospective associations between dairy intake and biomarkers of low-grade systemic inflammation or insulin resistance. All associations with inflammation were analysed for each biomarker of low-grade systemic inflammation and the pro-inflammatory score. Compared with similar studies^(24,37)^, results from the regression analyses are presented as adjusted least-square means (95 % CI) by sex-specific tertiles (low, medium and high intake) of the respective predictor, with *P*_trend_ values from models with the predictors as continuous variables.

Previously, individual outliers of every biomarker that significantly affected the normal distribution or regression modelling were winsorised, that is, the outliers were replaced by the closest sex-specific value corresponding to a normal distribution. The procedure involved IL-6 (1 %) and adiponectin (<1 %).

No stratification by sex was conducted as no significant interactions between dairy intake and sex for primary outcomes were observed in the analysis.

All basic models (model 1) included the exposure variable (intake of TD or each dairy type separately) during adolescence, sex and age at blood withdrawal in adulthood. For adjusted models (model 2), potential confounders were included individually and hierarchically if they significantly affected the regression coefficient of exposure by ≥10 % or predicted the outcome variable independently^(38,39)^. To ensure comparability, all pro-inflammatory score models (model 2) were identically adjusted. The same applies to the HOMA2-IR models. On the basis of the hierarchical examination of possible confounders, all adjusted models for all outcomes (model 2) included BMI in adulthood only.

To reduce potential bias, sensitivity analyses were conducted by excluding under-reported records. Dietary records were classified as ‘under-reported’ if the relationship between total energy intake and estimated BMR according to age- and sex-specific equations by Schofield^(40)^ was not plausible. Under-reported records were identified using the paediatric cut-offs by Sichert-Hellert^(41)^. This calculation resulted in the records of ten participants being identified as under-reported (2·7 %).

To exclude any possible bias of a short gestation period (<37 weeks) or low birth weight (<2500 g) on the outcome, further sensitivity analyses were performed wherein data from relevant participants were excluded (in total, *n* 20 participants, 5·3 %). In addition, sensitivity analyses were conducted by excluding participants with no data on smoking status (*n* 64, 17·0 %) and those with a known intolerance against dairy (*n* 13, 3·5 %). Because we could not determine whether the intolerance was a sensitivity or an allergy to dairy products, all thirteen participants with a general intolerance were excluded.

A further sensitivity analysis was conducted to consider the possible effect of dairy intake changes during adolescence^(22)^ that could have masked the effects of long-term dairy intake on the different outcomes. For this purpose, dietary data from early adolescence (boys, 9–12·5 years, and girls, 8–11·5 years) and late adolescence were considered in stratified analyses (boys, 12·5–16 years, and girls, 11·5–15 years).

## Results

Sample characteristics in adolescence and young adulthood of the participants (*n* 375) are shown in Tables 1 and 2. Approximately half of the participants were male (46·4 %). Participants and their mothers’ BMI values were within the normal range. Maternal employment amounted to >66 %. The median age at adolescence was 12 years (Table 1), and the median age at blood withdrawal was 21 years (Table 1). The median follow-up period between dietary records in adolescents and blood withdrawal in adulthood was 9·2 years. The daily median TD intake was 177·9 g/1000 kcal. Participants consumed more non-fermented than fermented, more low-sugar than high-sugar and more high-fat than low-fat dairy products. Non-fermented dairy had the highest median intake values in all tertiles (Table 4).

**Table 4:** Tertiles of dairy intake of 375 participants of the DONALD study in adolescents (1985–2019)

	Dairy intake (100 g/1000 kcal)*					
	Low intake		Moderate intake		High intake	
	Tertile 1		Tertile 2		Tertile 3	
	Median	25th, 75th percentiles	Median	25th, 75th percentiles	Median	25th, 75th percentiles
Total dairy	1·11	0·90; 1·27	1·78	1·64; 1·91	2·63	2·33; 2·96
Liquid dairy	0·29	0·16; 0·41	0·79	0·65; 0·93	1·62	1·31; 1·99
Solid dairy	0·54	0·39; 0·63	0·87	0·78; 0·97	1·26	1·16; 1·55
Fermented dairy	0·23	0·16; 0·28	0·45	0·39; 0·50	0·69	0·60; 0·86
Non-fermented dairy	0·64	0·46; 0·81	1·31	1·17; 1·44	2·04	1·84; 2·40
Low-fat dairy	0·11	0·07; 0·15	0·36	0·29; 0·51	1·28	0·96; 1·83
High-fat dairy	0·50	0·39; 0·64	1·07	0·89; 1·24	1·90	1·67; 2·27
Low-sugar dairy	0·61	0·46; 0·77	1·25	1·09; 1·38	1·99	1·77; 2·29
High-sugar dairy	0·24	0·17; 0·30	0·47	0·42; 0·52	0·77	0·65; 0·90

DONALD, DOrtmund Nutritional and Anthropometric Longitudinally Designed.

* Values refer to median (25th, 75th percentiles) of intake in the respective sex-specific tertile.

Overall, no association was observed between TD intake and the pro-inflammatory score. Similarly, no dairy type was associated with the pro-inflammatory score (Table 5). Grouping dairy intake into tertiles did not show any associations. The same applies to the examination of individual inflammation biomarkers (see online supplementary material, Supplementary Tables a–c).

**Table 5:** Prospective associations of dairy intake during adolescence with a pro-inflammatory score in young adulthood (*n* 375)

	Pro-inflammatory score						
	Tertile 1		Tertile 2		Tertile 3		
	Mean	95 % CI	Mean	95 % CI	Mean	95 % CI	*P*_trend_ *
Total dairy							
Model 1†	0·01	–0·09; 0·11	−0·10	–0·19; 0·00	0·09	–0·01; 0·19	0·28
Model 2‡	0·02	–0·07; 0·11	−0·07	–0·17; 0·02	0·04	–0·05; 0·14	0·80
Liquid dairy							
Model 1†	−0·01	–0·10; 0·09	−0·04	–0·14; 0·05	0·05	–0·05; 0·15	0·35
Model 2‡	0·01	–0·09; 1·00	−0·03	–0·12; 0·06	0·02	–0·07; 0·11	0·72
Solid dairy							
Model 1†	−0·05	–0·15; 0·05	0·06	–0·03; 0·16	−0·01	–0·11; 0·09	0·65
Model 2‡	−0·04	–0·13; 0·05	0·05	–0·04; 0·14	−0·01	–0·11; 0·08	0·91
Fermented dairy							
Model 1†	0·04	–0·05; 0·14	−0·01	–0·11; 0·08	−0·03	–0·13; 0·07	0·51
Model 2‡	0·06	–0·03; 0·15	0·00	–0·09; 0·09	−0·07	–0·16; 0·02	0·72
Non-fermented dairy							
Model 1†	0·02	–0·08; 0·11	−0·09	–0·19; 0·00	0·08	–0·02; 0·17	0·39
Model 2‡	0·02	–0·07; 0·11	−0·09	–0·18; 0·01	0·05	–0·04; 0·15	0·68
Low-fat dairy							
Model 1†	−0·02	–0·12; 0·08	0·00	–0·10; 0·10	0·02	–0·08; 0·11	0·16
Model 2‡	−0·02	–0·07; 0·11	0·01	–0·08; 0·10	−0·04	–0·13; 0·06	0·97
High-fat dairy							
Model 1†	0·01	–0·09; 0·11	−0·02	–0·12; 0·08	0·01	–0·09; 0·11	0·81
Model 2‡	−0·02	–0·11; 0·07	0·01	–0·09; 0·10	0·01	–0·09; 0·10	0·81
Low-sugar dairy							
Model 1†	−0·02	–0·12; 0·08	−0·04	–0·14; 0·06	0·06	–0·04; 0·16	0·30
Model 2‡	0·01	–0·08; 0·11	−0·03	–0·13; 0·06	0·01	–0·08; 0·11	0·91
High-sugar dairy							
Model 1†	−0·02	–0·12; 0·08	−0·01	–0·11; 0·08	0·04	–0·06; 0·13	0·79
Model 2‡	−0·06	–0·15; 0·03	−0·01	–0·10; 0·08	0·06	–0·03; 0·115	0·43

Model values are least-square means (95 % CI) for tertiles obtained from linear regression models.

* *P*_trend_ values are based on models using the continuous exposure variables.

† Adjusted for sex and age at blood withdrawal.

‡ Adjusted for sex, age at blood withdrawal as well as BMI in adulthood.

In addition, the analysis of TD intake and each dairy-type intake and insulin resistance showed no association (Table 6). No association was found when grouping dairy intake into tertiles.

**Table 6:** Prospective associations of dairy intake during adolescence with insulin resistance in young adulthood (*n* 371)

	HOMA2-IR§						
	Tertile 1		Tertile 2		Tertile 3		
	Mean	95 % CI	Mean	95 % CI	Mean	95 % CI	*P*_trend_ *
Total dairy							
Model 1†	0·39	0·33; 0·45	0·39	0·32; 0·45	0·39	0·33; 0·45	0·76
Model 2‡	0·40	0·34; 0·46	0·40	0·34; 0·46	0·36	0·31; 0·42	0·61
Liquid dairy							
Model 1†	0·40	0·33; 0·46	0·37	0·30; 0·43	0·40	0·34; 0·47	0·49
Model 2‡	0·40	0·34; 0·46	0·38	0·32; 0·44	0·38	0·32; 0·44	0·87
Solid dairy							
Model 1†	0·40	0·33; 0·46	0·42	0·36; 0·48	0·35	0·29; 0·41	0·60
Model 2‡	0·41	0·35; 0·47	0·41	0·35; 0·47	0·35	0·29; 0·41	0·25
Fermented dairy							
Model 1†	0·42	0·35; 0·48	0·34	0·28; 0·40	0·41	0·35; 0·47	0·61
Model 2‡	0·43	0·37; 0·49	0·34	0·28; 0·40	0·39	0·33; 0·45	0·65
Non-fermented dairy							
Model 1†	0·42	0·35; 0·48	0·36	0·30; 0·43	0·37	0·32; 0·45	0·91
Model 2‡	0·42	0·36; 0·48	0·37	0·31; 0·43	0·37	0·31; 0·43	0·73
Low-fat dairy							
Model 1†	0·37	0·31; 0·43	0·39	0·33; 0·46	0·40	0·34; 0·47	0·23
Model 2‡	0·39	0·33; 0·45	0·40	0·34; 0·46	0·37	0·31; 0·43	0·93
High-fat dairy							
Model 1†	0·40	0·34; 0·47	0·37	0·31; 0·43	0·39	0·33; 0·46	0·38
Model 2‡	0·39	0·33; 0·45	0·38	0·33; 0·44	0·39	0·33; 0·45	0·63
Low-sugar dairy							
Model 1†	0·37	0·31; 0·44	0·40	0·31; 0·43	0·42	0·36; 0·49	0·28
Model 2‡	0·39	0·33; 0·45	0·37	0·31; 0·43	0·40	0·34; 0·46	0·94
High-sugar dairy							
Model 1†	0·44	0·38; 0·50	0·38	0·32; 0·45	0·34	0·28; 0·41	0·11
Model 2‡	0·42	0·36; 0·48	0·39	0·33; 0·45	0·36	0·30; 0·41	0·20

HOMA2-IR, Homeostasis Model Assessment Insulin Resistance.

Model values are least-square means (95 % CI) for tertiles obtained from linear regression models. Outcome variables were log-transformed.

* *P*_trend_ values are based on models using the continuous exposure variables.

† Adjusted for sex and age at blood withdrawal.

‡ Adjusted for sex, age at blood withdrawal as well as BMI in adulthood.

§ HOMA2-IR.

Sensitivity analyses excluding under-reported records confirmed our null results for both the pro-inflammatory score and insulin resistance (data not shown). Our results also remained the same after the exclusion of participants with a low birth weight or a short duration of pregnancy, participants without information on smoking status or participants with intolerance to dairy (data not shown). Stratifying the data into records from early adolescence and records from late adolescence also had no consequence and confirmed the reliability of the results for both outcomes.

## Discussion

This study examined the long-term association of habitual dairy intake in healthy adolescents with inflammation biomarkers and insulin resistance in young adulthood. The daily median TD intake of 177·78 g/1000 kcal in our sample is approximately 30 % below that is specified by the German Food-Based Dietary Guidelines for adolescents^(42)^. This is in accordance with the representative EsKiMo II study, which analysed the dietary behaviour of 2644 children and adolescents in Germany between 2015 and 2017. In this study, approximately half of German children and adolescents aged 6–17 years did not achieve the recommended dairy intake^(43)^. We did not find any association between dairy or different types of dairy intake and pro-inflammatory score or insulin resistance. Accordingly, our study shows that missing associations observed in previous cross-sectional studies in healthy children can also be confirmed in the long term, considering adolescent dairy intake and blood parameters in young adulthood^(18,44,45)^. To the best of our knowledge, this is the first study to examine different types of dairy in this context.

The absence of any association with the pro-inflammatory score suggests that dairy intake in adolescence is not of major longer-term relevance for low-grade systemic inflammation among young adults. Our findings are in line with those of a systematic review by Bujtor *et al.* ^(19)^ on the associations of dietary intake with single biomarkers of inflammation (C-reactive protein or IL-6) in healthy children and adolescents. However, in this review, only seven studies examined dairy intake, of which only five included healthy participants. A cross-sectional study by Abreu *et al.* ^(46)^ suggested an inverse association between TD or milk intake and serum IL-6 concentrations among normal-weight adolescents; moreover, adolescents in the second tertile of yoghurt intake showed IL-6 lower levels than that of those in the first tertile. In our analysis, we did not find a difference between intake tertiles for the pro-inflammatory score and any biomarker of inflammation. However, we considered fermented dairy in general terms and not yoghurt intake specifically. Furthermore, we examined only the long-term associations and not the short-term ones.

In a systematic review, Bordoni *et al.* ^(14)^ developed a pro-inflammatory score from various individual biomarkers, comparable to the inflammation score used in the present analysis. The authors summarised the anti-inflammatory activity of dairy intake in adults with metabolic disorders and the pro-inflammatory activity in adults allergic to cow’s milk. A stratified consideration of different types of dairy in this analysis indicated a weak anti-inflammatory activity of low-fat and high-fat as well as fermented dairy in participants who were not allergic to dairy products^(14)^. Our analyses did not confirm this anti-inflammatory association even after excluding participants with a general intolerance to dairy (data not shown). However, in our sample, only six participants (3·5 %) reported such intolerance. In addition, the analyses by Bordoni *et al.* ^(14)^ were also based on analyses among adults. Further research in this context is required. A narrative review^(47)^ resumed that most SFA activate pro-inflammatory biomarkers. Which combination of nutrients results in a neutral or inverse association between dairy intake and inflammation remains to be clarified.

With regard to the relationship between dairy intake during adolescence and insulin resistance in young adulthood, our results are not in line with those of the analysis of the ‘Nurses’ Health Study II’ cohort by Malik *et al.* ^(48)^, in which higher dairy intake during adolescence was associated with a lower risk of type 2 diabetes in adulthood. Our analyses did not find an association between TD intake and dairy-type intake and insulin resistance. The different results can probably be attributed to different methods. Malik *et al.* ^(48)^ investigated inflammatory biomarkers exclusively in middle-aged women, whereas our study took both sexes into account. However, tests for the interaction of inflammatory markers with sex in our sample indicated no difference. In addition, the risk of inflammation among nurses may be increased because of shift work^(49)^. Additionally, Malik *et al.* ^(48)^ applied a FFQ to assess participants’ diet during high school, which carries the risk of recall bias. However, the sample size in Malik *et al.* ^(48)^ was much larger than that in our study.

Our results also did not confirm the findings reported in a systematic review and meta-analysis of prospective studies^(50)^ or in a systematic review and meta-analysis of randomised clinical trials^(51)^ that suggested a beneficial effect of low-fat dairy on HOMA2-IR. They assumed that Ca, vitamin D, casein and whey proteins in low-fat dairy are potential regulators of body fat, waist circumference and insulin resistance. However, we could not find any associations independent of the type of dairy. High levels of adipose tissue were assumed to result in the development of insulin resistance^(52)^. In a previous study based on our sample, higher TD intake led to a more favourable body composition in the long term; however, higher intake of low-fat dairy showed no association^(23)^. However, our sample is characterised by a rather low BMI compared with the general population in Germany^(53,54)^.

Some strengths and limitations of this analysis of the DONALD study must be discussed. The prospective design and repeatedly collected detailed dietary measurements allow the investigation of long-term associations between adolescent habitual dairy intake including subtypes and adult health outcomes^(20)^. The continuously updated in-house nutrient database LEBTAB allows the consideration of different types of dairy according to composition and processing methods^(21)^. The main limitation of our sample is the over-representation of families with a high socio-economic background in the DONALD study, which limits the generalisability of our results^(20)^. Although our sample was not very large, and relatively young and healthy, previous analyses showed that significant associations, for example, between dietary intake and risk factors for type 2 diabetes, could still be shown with the data from the DONALD study^(26)^.

Food grouping in our study is both a strength and a limitation. The dietary assessment method and food composition database allow the aggregation of several diverse food groups. However, some of these subgroups overlap to allow the investigation of possible associations with health (e.g. fermentation and fat content). A finer subdivision would have led to very low intake quantities. The underlying classification has already been used in our previous publications^(22,23)^ and allows an overall interpretation of the results.

In addition, we cannot reject the possibility of under-reporting. Under-reported records were not generally excluded from the main analyses because this method only identifies under-reported energy intake and unselective under-reporting of individual foods^(55)^. However, our sensitivity analyses, excluding energy under-reports, showed similar results to those of the main analyses.

### Conclusions

Our results indicated the absence of any associations between dairy intake and pro-inflammatory score or insulin resistance in young adulthood. Thus, the habitual intake of individual types of dairy during childhood and adolescence does not influence these metabolic risk factors in the long term. Restrictions on dairy intake for healthy children and adolescents appear redundant in terms of diabetes risk reduction.

## Supporting information

Hohoff et al. supplementary material Hohoff et al. supplementary material

## Supplementary material

For supplementary material accompanying this paper, visit https://doi.org/10.1017/S1368980024000624.
