# Supplementary material for: The association between dairy intake in adolescents on inflammation and risk markers of type 2 diabetes during young adulthood: results of the DONALD study
Source: Public Health Nutr. 2024 Mar 13;27(1):e91. doi: 10.1017/S1368980024000624 (PMC10966841; doi:10.1017/S1368980024000624)
Supplement: Hohoff et al. supplementary material [file S1368980024000624sup001.docx]

**Supplementary Table a:** Prospective associations of dairy intake during adolescence with hsCRP levels in young adulthood (n = 375)

**Supplementary Table b:** Prospective associations of dairy intake during adolescence with IL-6 and IL-18 levels in young adulthood (n = 375)

**Supplementary Table c:** Prospective associations of dairy intake during adolescence with leptin and adiponectin in young adulthood (n = 375)
